# Association between Polymorphism of the Vitamin D Metabolism Gene CYP27B1 and HLA-B27-Associated Uveitis. Is a State of Relative Immunodeficiency Pathogenic in HLA B27-Positive Uveitis?

**DOI:** 10.1371/journal.pone.0062244

**Published:** 2013-04-17

**Authors:** Gernot Steinwender, Ewald Lindner, Martin Weger, Sophie Plainer, Wilfried Renner, Navid Ardjomand, Yosuf El-Shabrawi

**Affiliations:** 1 Department of Ophthalmology, Auenbrugger University Graz, Graz, Austria; 2 Department of Ophthalmology, Klinikum Klagenfurt, Klagenfurt, Austria; 3 Clinical Institute of Medical and Chemical Laboratory Diagnostics, Medical University Graz, Graz, Austria; Medical University Graz, Austria

## Abstract

**Objective:**

Polymorphisms of the vitamin D metabolism gene CYP27B1 showed associations with multiple autoimmune diseases. The aim of this study was to investigate a possible association between the rs703842 A>G polymorphism of the CYP27B1 gene and HLA-B27-associated uveitis.

**Design:**

One hundred fifty-nine patients with HLA-B27-associated uveitis, 138 HLA-B27-negative controls and 100 HLA-B27-positive controls were recruited for this retrospective case-control study. Main outcome parameters were genotype distribution and allelic frequencies determined by polymerase chain reaction.

**Results:**

Carriers of the rs703842G allele were found significantly more often in patients with HLA-B27-associated uveitis than in HLA-B27-positive controls (*p* = 0.03). Between patients and HLA-B27-negative controls no significant difference in the genotype distribution of the rs703842 A>G polymorphism was found (*p* = 0.97).

**Conclusions:**

Our data suggest that the rs703842 A>G polymorphism may play a role in HLA-B27-associated uveitis.

## Introduction

The by far most common type of uveitis is acute anterior uveitis (AAU). It is an important cause of visual impairment in western populations [1], [2]. About 50% of all cases of AAU are associated with a positive Human leukocyte antigen (HLA) B27 haplotype [3], [4]. HLA-B27-associated AAU represents a well-defined clinical entity and occurs usually unilateral, but both eyes may be affected sequentially.

HLA-B27 is closely linked not just with AAU but a spectrum of seronegative spondyloarthropathies (SpA) [5]. Development of these systemic inflammatory diseases is seen in almost half of patients suffering from HLA-B27-associated AAU [3]. The strongest disease association of HLA-B27 positivity is demonstrated in ankylosing spondylitis (AS) with around 90% of patients possessing the HLA-B27 haplotype. Other HLA-B27-associated systemic inflammatory diseases include reactive arthritis, psoriatic arthropathy, inflammatory bowel disease, and undifferentiated SpA [6].

Since only 2% of the individuals carrying the HLA-B27 haplotype (7–8% in Caucasians) will eventually develop SpAs or an AAU [2], [7], [8], additional environmental and genetic factors contributing to disease development have been suggested. In particular, bacterial triggers have been shown to play a critical role in the development of HLA-B27^+^-associated AAU and SpA. After genitourinary or gastrointestinal tract infection with Gram-negative bacteria microbe-derived antigens may trigger a CD8-restricted T lymphocyte immune response that cross-reacts with self-tissue antigens, resulting in an autoimmune tissue inflammation [3], [9]. In addition, it has been proposed that the HLA-B27 haplotype plays an immunmodulatory role. Its presence has been correlated to an enhanced intracellular invasion or impaired intracellular elimination of gram-negative bacteria [10]–[13]. Thus it is feasible that the reduced ability to clear off intracellular antigens, as a result of the down-regulated inflammatory response, may result in the induction of chronic auto-inflammatory disease in HLA-B27^+^ individuals.

The established association between vitamin D deficiency and many autoimmune diseases [14]–[16] encouraged us to investigate a possible role of the vitamin D metabolism gene CYP27B1 (cytochrome P450 family 27 subfamily B peptide 1) in the development of HLA-B27-associated AAU. Large genome-wide association studies (GWAS) detected SNPs (single nucleotide polymorphisms) within this gene associated with type 1 diabetes [17], [18], Hashimotòs thyroiditis, Graves` disease [19] and multiple sclerosis (MS) [20], [21]. CYP27B1 encodes the enzyme 25-hydroxy-vitamin D-1 alpha hydroxylase, which hydroxylates the precursor of vitamin D3, 25-OHD_3_, in its more bioactive form, 1,25(OH)_2_D_3_. Besides regulating calcium metabolism through binding to the vitamin D receptor (VDR), vitamin D3 also plays an important role in the innate and adaptive immune system [22]. In particular, 25-OHD_3_ stimulates the expression of cathelicidin, an antibacterial peptide with critical influence on innate immune defense against invasive bacterial infection [23]. Furthermore, 1,25(OH)_2_D_3_ suppresses the adaptive immune response by enhancing the development of anti-inflammatory T helper cells type 2 (Th2) as well as inhibiting the development of T helper cells type 1 (Th1) [24], [25].

In a recent GWAS rs703842 was identified as the strongest MS-associated polymorphism of the CYP27B1 gene [20]. The G-allele of rs703842 was shown to be significantly associated with lower levels of 25-OHD_3_ in a Canadian twin-study [26]. To the best of our knowledge CYP27B1 polymorphisms have not yet been studied in HLA-B27-associated AAU. Therefore, the purpose of this study was to investigate a possible association between the rs703842 A>G polymorphism and HLA-B27-associated AAU. In that context our group found significant associations with gene polymorphisms of monocyte chemoattractant protein-1 (MCP-1) and tumor necrosis factor-α (TNF-α) promoter [27], [28]. The influence of those SNPs on AAU-susceptibility might be explained with a state of relative immunodeficiency in HLA-B27-positive individuals, leading to a prolonged bacterial persistence. This notion would be further supported in case of a correlation of HLA-B27-associated AAU and the G-allele of rs703842.

## Materials and Methods

In the present retrospective case-control study 159 patients with acute HLA-B27-associated uveitis, 138 HLA-B27-negative controls and 100 HLA-B27-positive controls were enrolled. Written informed consent was obtained from all participants prior to enrolment. The study was conducted in compliance of the principles of the Declaration of Helsinki and has been approved by the Ethics Committee of the Medical University Graz.

The following data were collected from all participants: gender, age at presentation, age at onset of anterior uveitis, diagnosis of associated systemic disease, number and duration of flares, and severe ocular complications (vitreous inflammation≥2+cells, cataract ≥2+opacity, secondary glaucoma, clinically significant macular edema as visualized by optic coherence tomography or fluorescein angiography). Patients with Fuchs` heterochromic iridocyclitis, sarcoidosis, or any history of malignancy were excluded from our investigation. All participants underwent an examination by a rheumatologist, including radiographs of the sacroiliac joints and the spine in presence of symptoms compatible with spondyloarthropathy.

The control cohort included 138 random, unrelated, healthy individuals who visited our clinic for reasons other than ocular inflammation. Subjects positive for HLA-B27, or with any history of ocular inflammation, autoimmune diseases, lower back pain or malignancy were not included as HLA-B27-negative control patients. 100 HLA-B27-positive, healthy, unrelated blood donors, whose DNA was provided by the Department of Blood Serology and Transfusion Medicine, served as the HLA-B27-positive control group.

### Laboratory methods

Blood samples from all subjects were collected in vaccutainers containing EDTA and stored at −20°C. Genomic DNA was isolated using the QIAmp Blood Mini kit (Quiagen GmbH, Hilden, Germany). Genotyping was performed by high-resolution melting (LightCycler® 480 System, Roche Diagnostics, Vienna, Austria) following a protocol previously described [29]. Gene scanning software version 1.5 (Roche Applied Science, Mannheim, Germany) was used to determine sequence variations.

### Statistics

SPSS 15.0 for Windows (SPSS Inc., Chicago, IL) was used to analyze data. Genotype and allele frequencies were compared between patients and controls using the χ2 test. Logistic regression analysis was performed to calculate Odds ratios (OR) and 95% confidence intervals (95% CI). A *P*-value<0.05 was considered statistically significant.

## Results

Baseline characteristics of patients and controls are presented in Table 1, while the clinical characteristics of the patients are shown in Table 2. Ankylosing spondylitis (AS), the most common systemic manifestation in our cohort, was found in 71 (44.7%) out of 159 patients. 24 (15.1%) patients suffered from undifferentiated spondyloarthritis, 6 (3.8%) from reactive arthritis (ReA), 15 (9.4%) from psoriatic arthritis (PsA), and 1 (0.6%) patient suffered from Crohn`s disease.

**Table 1:** Demographic Characteristics of Patients and Controls.

	Patients with HLA-B27-Associated AAU (n = 159)	HLA-B27-Negative Controls (n = 138)	HLA-B27-Positive Controls (n = 100)
Male	88 (55.3)	97 (70.3)	49 (49.0)
Female	71 (44.7)	41 (29.7)	51 (51.0)
Mean Age±SD (yrs)	44.8±14.3	35.3±12.5	38.2±4.2

AAU = Acute Anterior Uveitis

SD = Standard Deviation

Values are n (%) unless otherwise indicated. The mean age for the patient group states the age of onset of the disease.

**Table 2:** Baseline Ocular and Systemic Parameters.

Ocular parameters:	
One eye affected	96 (60.4)
Both eyes alternating	54 (34.0)
Both eyes concomitant	9 (5.6)
Mean number of flares±SD	7.19±9.24
Mean duration of flares±SD (weeks)	4.09±2.74
Mean duration between flares±SD (months)	20.95±18.31
Secondary cataract	17 (10.7)
Secondary glaucoma	5 (3.1)
Posterior segment inflammation	31 (19.5)
Macular edema	21 (13.2)
Systemic parameters:	
Ankylosing spondylitis	71 (44.7)
Juvenile idiopathic arthritis	1 (0.6)
Undifferentiated spondylarthritis	24 (15.1)
Reactive arthritis	6 (3.8)
Crohńs disease	1 (0.6)
Psoriatic arthritis	15 (9.4)
Overall systemic manifestation	118 (74.2)

SD = Standard Deviation

Values are n (%) unless otherwise indicated.

Table 3 shows the genotype distribution of the rs703842 A>G polymorphism in patients with HLA-B27-associated AAU and the two control groups. All allele and genotype frequencies were in Hardy-Weinberg-equilibrium. Carriers of the rs703842G allele were found significantly more often in patients with HLA-B27-associated AAU compared to healthy HLA-B27-positive individuals (OR = 0.62, 95% CI 0.41–0.94; p = 0.03). As the CYP27B1 gene is located on chromosome 12 and the gene for MHC-class I molecule HLA-B27 lies on chromosome 17 a linkage in the inheritance of these two genes is rather unlikely.

**Table 3:** Distribution of the rs703842 A>G Gene Polymorphism in Patients and Controls.

	Patients with HLA-B27-Associated AAU (n = 159)	HLA-B27-Negative Controls*(n = 138)	HLA-B27-Positive Controls**(n = 100)
GG	14(8.8)	6(4.3)	5(5.0)
GA	66(41.5)	70(50.7)	31(31.0)
AA	79(49.7)	62(44.9)	64(64.0)

AAU = Acute Anterior Uveitis, OR = Odds Ratio, CI = Confidence Interval, Values are n (%) unless otherwise indicated.

* HLA-B27-associated patients vs. HLA-B27-negative control subjects ***p*** = 0.97* (OR = 1.01, 95% CI 0.70–1.46)

** HLA-B27-associated patients vs. HLA-B27-positive control subjects *p* = 0.03** (OR = 0.62, 95% CI 0.41–0.94)

No statistically significant difference in the distribution of the rs703842 A>G polymorphism was observed between HLA-B27-positive and HLA-B27-negative controls (p = 0.54). The frequency of the minor allele also did not significantly differ between patients and healthy HLA-B27-negative subjects (p = 0.97).

There was no significant association between rs703842 A>G genotypes and recurrence of uveitis flare, and we did not find any association between rs703842 and underlying systemic diseases in our patient cohort. No significant difference in the genotype distribution of rs703842 in AAU-patients suffering from AS compared to AAU-patients without AS was observed (OR = 0.97, 95% CI 0.59–1.59; p = 0.89).

## Discussion

In the present study, we investigated the association of the CYP27B1 gene polymorphism rs703842 A>G with HLA-B27-associated AAU in a central European population. We observed a significant higher prevalence of the G-allele in HLA-B27-associated AAU patients compared to healthy HLA-B27-positive controls.

Recently, immunomodulatory actions of vitamin D and an association between vitamin D deficiency and many autoimmune diseases have been reported [14]–[16]. Besides suppressing the adaptive immune response by enhancing the development of anti-inflammatory Th2-cells and inhibiting the development of Th1-cells [24], [25], vitamin D3 also plays an important role in the innate immune response. It has been shown that vitamin D supplementation in vivo can enhance the expression of cathelicidin, an antibacterial peptide [23]. Cathelicidin was identified several years ago as a target for transcriptional regulation by 1,25(OH)2D3-liganded vitamin D receptor (VDR) [30], [31] and has a critical influence on the innate immune defense against invasive bacterial infection [23].

Lower levels of 25-OHD_3_, which were reported to be significantly associated with the G-allele of rs703842 [26], are thought to result in a reduced expression of the antibacterial protein cathelicidin, with a consecutive impaired pathogen elimination [23]. The finding of our study, where we find a higher prevalence of the G-allele in HLA-B27-associated AAU patients, suggests that a state of a relative immune deficiency in HLAB27^+^ patients, is pathognomoic in HLA B27^+^ associated diseases, is in accordance with those of a recent gene expression profiling study [32]. Duan et al. described gene expression patterns in white blood cells in AS patients, an immunosuppressive phenotype [32].

Thus, a possible explanation for the increased AAU-susceptibility in HLA-B27-positive individuals harboring the minor G-allele is a further reduction of the already impaired ability of HLA-B27-positive individuals to clear off intracellular pathogens [10]–[13] through an additional immunosuppressive function of the investigated rs703842-SNP, ultimately leading to chronic autoinflammatory response of the immune system.

The results of the present study, also recapitulate previous findings of our group and thus supports the relevance of the investigated rs703842-SNP. Recently, we identified polymorphisms in the MCP-1 gene, which influence the susceptibility for HLA-B27-associated AAU via the aforementioned inefficient clearance of infectious agents [27]. We were also able to show that SNPs of the TNF-α promoter, leading to an increased transcription of TNF-α, an important factor in early stages of the innate immune response, had a protective effect against HLA-B27-associated AAU [28].

This is the first study to examine the role of the CYP27B1 gene in HLA-B27-associated AAU. The limitations of this study are those inherent to any other retrospective study. Genetic factors, however, unlike many other biologic parameters, are not influenced during lifetime.

In conclusion, our data suggest an association between the rs703842 A>G polymorphism and the risk for HLA-B27-associated AAU. Further research will be required to elucidate the underlying mechanisms of pathogenesis more precisely.
